# First Record of the Genus *Aprivesa* Melichar (Hemiptera: Fulgoromorpha) from South India, with Description of One New Species

**DOI:** 10.3897/zookeys.81.816

**Published:** 2011-02-18

**Authors:** Cui-Ping Bu, Ai-Ping Liang

**Affiliations:** Key Laboratory of Zoological Systematics and Evolution, Institute of Zoology, Chinese Academy of Sciences, 1 Beichen West Road, Chaoyang District, Beijing 100101, P.R.China

**Keywords:** Ricaniidae, Fulgoroidea, taxonomy, biodiversity, distribution

## Abstract

Aprivesa unimaculata **sp. n.** (Hemiptera: Fulgoromorpha: Ricaniidae) is described and illustrated from Coorg, south India. This represents the first record of the genus Aprivesa Melichar from India and the fourth known species of Aprivesa. The new taxon greatly extends the range of the genus Aprivesa, which was previously known as an endemic Australian genus. A checklist of all known species of the Ricaniidae from India and keys to all the known genera of the Ricaniidae from India and all species in the genus are provided.

## Introduction

Ricaniidae is one of the larger families of the Fulgoroidea, comprising more than 450 described species in approximately 46 genera (Montrouzier 1861, Melichar 1898a, b, 1923, Metcalf 1955, Fennah 1968, 1969, 1971, Williams and Fennah 1980, Shcherbakov 2006, Fletcher 2008, Bu et al. 2010). Members of the group are distributed widely in the Afrotropical, Australian, Indo-Malayan and Oceania regions, and primarily around the tropics (Metcalf 1955, Miklos 1975). Most of the species are of little economic importance but a few are major agricultural pests, such as Ricania speculum, Pochazia sublimate and Scolycopa australis (Charles 1998, Fletcher 1979a, b, 2008, Luo 2003).

The ricaniid fauna of India remains inadequately studied and there is still much basic taxonomic work to be done on the group. To date, 28 species in 9 genera from the Ricaniidae are described or recorded from India (Distant 1906, 1909, 1916, Metcalf 1955, Ghauri 1973). The number of the described species likely represents only a small fraction of the actual diversity of the whole Indian ricaniid fauna considering the vast territory and various complex habitats of India.

The genus Aprivesa was established by Melichar (1923) for Privesa exucta Melichar, 1898 from Australia. Muir (1931) described the second species of the genus Aprivesa varipennis from Western Australia. More recently, Fletcher (2008) transferred Privesa pronotalis Walker, 1917 into the genus. Until now, Aprivesa contains 3 known species and they are all endemic to Australia.

While sorting and identifying the Ricaniidae from material in the Department of Entomology Insect Collection, North Carolina State University, Raleigh, NC, USA, we found a new species of Aprivesa from south India. The new species represents the first record of Aprivesa in India, and its discovery has broadened our knowledge of the morphology and biogeography of the genus. In this paper, we redescribe the genus Aprivesa and describe and illustrate the new species from south India. A key is given for the separation of the known species in Aprivesa. A checklist of all known species of the Ricaniidae from India and a key to all the known genera of the Ricaniidae from India are also provided.

## Materials and methods

The specimens studied in the course of this work are deposited at the Department of Entomology Insect Collection, North Carolina State University, Raleigh, NC, USA (NCSU).

Specimens used for dissection were cleaned in 10% KOH at room temperature for ca. 12 hours, rinsed in distilled H2O, stained by methylrosanilinium chloride (a clinical solution, comprising methyl violet, ethanol and purified water) to highlight the internal thin and transparent membranous parts, and then transferred to glycerol for examination. Morphological characters were observed with a Zeiss Stemi SV 11 optical stereomicroscope and were illustrated with the aid of a drawing tube attached to the microscope. Measurements were made with the aid of an eyepiece micrometer.

The following abbreviations are used in the text, BL: body length (from apex of cephalic process to tip of fore wing) and FWL: fore wing length.

The morphological terminology followed is that of Bu et al. (2010).

## Taxonomy

### Checklist of the Ricaniidae species from India

**Ricaniidae Amyot & Serville, 1843**

Apachnas Distant, 1909

Apachnas nobilis Distant, 1909. India (Madras)

Aprivesa Melichar, 1923

Aprivesa unimaculata sp. n. India (Coorg)

Euricania Melichar, 1898

Euricania ocellus (Walker, 1851). India (Assam, Sikkim)

Pochazia Amyot & Serville, 1843

Pochazia antica (Gray, 1832). India (Tamil Nadu)

Pochazia atkinsoni Distant, 1906. India (Sikkim)

Pochaziaconfusa Distant, 1906. India (Assam)

Pochazia guttifera Walker, 1851. India (Assam, Darjeeling, Sikkim)

Pochazia interrupta Walker, 1851. India (Assam, Malabar Coast)

Pochazia sinuata Stål, 1865. Northern India

Pochazia transversa Melichar, 1898. India (Darjeeling)

Ricania Germar, 1818

Ricania apicalis (Walker, 1851). India (Assam, Sikkim)

Ricania bicolorata Distant, 1906. India (Madras)

Ricania coorgensis Distant, 1916. India (Coorg)

Ricania fenestrata (Fabricius, 1775) India (Coorg, Kerala, MadrasTravancore, Trivandrum)

Ricania fumosa (Walker 1851). India (Assam)

Ricania marginalis (Walker 1851). India (Assam, Bombay, Coorg)

Ricania simulans (Walker, 1851). Northern India

Ricania speculum (Walker 1851). India (Assam, Madras, Nilgiri Hills, Sikkim, Trivandrum)

Ricania stupida (Walker, 1857). India (Assam)

Ricania taeniata Stål, 1870. India

Ricania zebra Distant 1906. India (Assam)

Ricanoides Zia, 1935

Ricanoides flabellum (Noualhier, 1896). India (Assam)

Ricanoptera Melichar, 1898

Ricanoptera inculta Melichar, 1898. India (Assam, Nicobar Islands, Great Nicobar)

Ricanoptera polita Melichar, 1898. India (Nicobar Island, little Nicobar)

Ricanula Melichar, 1898

Ricanula pulverosa (Stål, 1865). India (Assam)

Ricanula stigma (Walker, 1851). India (Assam, Nicobar Islands)

Scolypopa Stål, 1859

Scolypopa confinis (Distant, 1906). India (Bombay, Coorg, Madras )

Scolypopa delecta (Melichar, 1898). India (Bombay)

### Key to genera of Ricaniidae from India

**Table:** 

1	Forewing quadrate, with costal and sutural margins subparallel (Figs 9–10, 13)	Aprivesa Melichar
–	Forewing more or less triangular (Figs 1–5, 8)	2
2	Forewing with sparse longitudinal veins, costal cell without transverse veinlets (Figs 1–2)	3
–	Forewing with dense longitudinal veins, costal cell with transverse veinlets (Figs 3–5, 8, 13)	4
3	Forewing with radial vein branched before pterostigma (Fig. 1)	Euricania Melichar
–	Forewing with radial vein not branched before pterostigma (Fig. 2)	Apachnas Distant
4	Forewing large, with apical angle prominent, apical margin longer than claval suture (Fig. 3)	Pochazia Amyot & Serville
–	Forewing relatively small, with apical angle rounded, apical margin nearly as long as claval suture	5
5	Forewing with cells on basal portion with numerous anastomosed crossveins	6
–	Forewing with cells on basal portion without anastomosed crossveins	8
6	Mesonotum with lateral carinae slightly, inwardly and anteriorly curved and bell-like (Fig. 6)	Ricanoides Zia
–	Mesonotum with lateral carinae distinctly, inwardly and anteriorly curved and angle-like (Fig. 7)	7
7	Forewing with precostal area with dense transverse veinlets, costal margin distinctly convex near base (see Yang 1989: 192, Fig. 11D)	Ricania Germar
–	Forewing with precostal area with sparse transverse veinlets, costal margin slightly convex near base (Fig. 8)	Ricanula Melichar
8	Forewing narrower with costal margin/apical margin ratio>1.4 (Fig. 4)	Scolypopa Stål
–	Forewing wider with costal margin/apical margin ratio<1.4 (Fig. 5)	Ricanoptera Melichar

**Figures 1–8.**
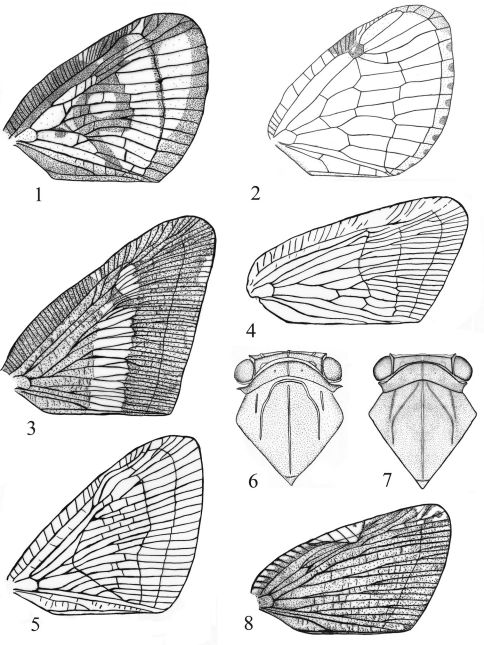
**1** Euricania ocellus (Walker, 1851) **2** Apachnas nobilis Distant, 1909 **3** Pochazia confusa Distant, 1906 **4** Scolypopa delecta (Melichar, 1898) **5** Ricanoptera inculta Melichar, 1898 **6** Ricanoides flabellum (Noualhier, 1896) **7** Ricania speculum (Walker, 1851) **8** Ricanula pulverosa (Stål, 1865) Notes: Fig. 1 quoted from Xu et al. (2006), Figs 3, 5, 8 quoted from Melichar (1898a), Fig. 4 quoted from Distant (1906).

### Genus Aprivesa

Melichar, 1923

Aprivesa Melichar 1923: 144. **Type species.**Privesa exutaMelichar 1898b, designated by Melichar 1923: 144.

#### Redescription.

General colour ochraceous or fuscous. Vertex and most part of frons usually pale brown or dark brown. Pronotum brown. Mesonotum usually fuscous. Legs pale yellow or brown. Forewing brown to fuscous. Hindwing hyaline, pale brown.

**Figures 9–10.**
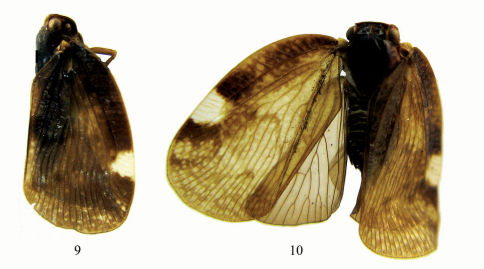
Habitus of Aprivesa unimaculatasp. n. **9** ♂, south India, lateral view **10** ♀, south India, dorsal view.

**Figures 11–14. F3:**
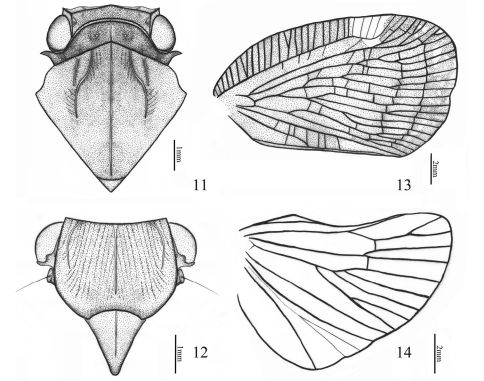
Aprivesa unimaculatasp. n. **11** head (♂), pronotum and mesonotum, dorsal view **12** head (♂), ventral view **13** fore wing (♂) **14** hind wing (♂).

Head (Figs 9–12, see Fletcher 2008: 112, Figs 15–17) large. Vertex broad and narrow, nearly rectangular in outline; distinctly separated from the frons by a transverse carina, lateral margins ridged and nearly parallel, posterior margin archedly concave; shorter than pronotum at midline; disk planar with some faint wrinkles. Frons oblique, broader than long, with central, sublateral and lateral carinae; lateral margins carinate and strongly elevated, with a slight outward bulge at mid-length, converging below level of antennae to apex. Clypeus narrower than frons, convex medially, shallowly inserted, lateral marginal areas depressed, with central longitudinal carina. Rostrum with subapical segment just surpassing meso-trochanters, apical segment attaining post-trochanters. Eyes oval. Ocelli small, situated between eye and base of antennae. Antennae short, scape ring-liked; pedicel subglobose, about 2 times as long as scape; flagellum setaceous, basely expanded.

Pronotum (Figs 9–11) narrow, with median longitudinal carina, punctuated beside central carina; disk slightly sloping laterally, hind margin centrally distinctly arched anteriorly. Mesonotum (Figs 9–11) large, triangular and convex, with 3 carinae: central carina straight; lateral carinae inwardly and anteriorly curved, nearly parallel on anterior margin, each bifurcating outwardly near middle in a straight longitudinal carina. Forewing (Figs 9–10, 13, see Fletcher 2008: 110–112, Figs 13–15;) quadrate, with costal and sutural margins subparallel; apical margin convex, shorter than claval suture; precostal area at middle broader than costal cell, with transverse veinlets dense; three veins emanating from basal cell, R and Sc nearly parallel, the radial veins originating from a common point on the basal cell; M leaving basal cell as a single short stem but forking in more than length of basal cell; Cu1 with four or five branches just before the apical margin; subapical line complete; claval veins uniting near middle of clavus, common claval vein entering commissural margin, clavus with many transverse veinlets. Hindwing (Figs 10, 14, see Fletcher 2008: 108, Fig. 2) small, anterior margin strongly sinuate; Sc short, unforked, R with three or four branches, M with two or three branches, Cu1 with more than four branches; transverse veinlets including only R-M and M-Cu. Legs moderately long; hind tibiae with 2 lateral black-tipped spines.

Female and male genitalia. See description of Aprivesa unimaculata sp. n. below.

**Figures 15–18. F4:**
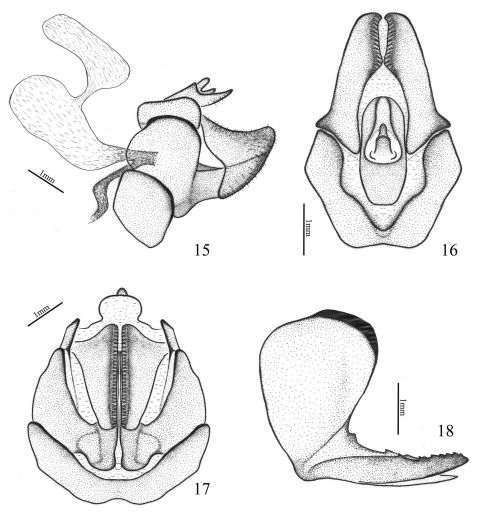
Aprivesa unimaculatasp. n. **15** genitalia (♀), lateral view **16** anal tube (♀), dorsal view **17** genitalia (♀), ventral view **18** gonopophyses VIII (♀), lateral view.

**Figures 19–23.**
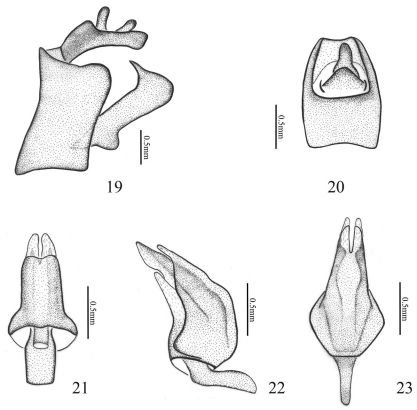
Aprivesa unimaculatasp. n. **19** genitalia (♂), lateral view **20** anal tube (♂), dorsal view **21** aedeagus, dorsal view **22** aedeagus, lateral view **23** aedeagus, ventral view.

#### Biology.

As with many ricaniid planthopper species, no biological data are currently available for species of Aprivesa, except that Aprivesa exuta was collected on Melaleuca quinquenervia (Fletcher 2008).

#### Distribution.

Australia, India.

#### Remarks.

Aprivesa is distinguished from other genera in Ricaniidae by the shape of frons and wing, the wing venation, and the minutiae of the male genitalia.

Species of Aprivesa are similar to those of Privesa Stål. But Aprivesa can be separated from Privesa by the lateral margins of the frons with a slight outward bulge below the antennae and the forewing with two radial veins originating from a common point on the basal cell (Fletcher 2008). In addition, the genus Privesa is distributed primarily in the Afrotropical region. Although the genus Aprivesa was an Australian endemic before, the finding of the new species in south India greatly extends the range of the genus Aprivesa. The similar distribution pattern is seen in another ricaniid genus Scolypopa. Most of Scolypopa species are found in the Australian region; but three distinct species of Scolypopa are distributed in the Indo-Malayan region (Metcalf 1955, Fletcher 1979a, b, 2008).

#### Key to species of genus Aprivesa.

**Table:** 

1	Forewing pale dull ochraceous, with dark mottlings; precostal area at middle 1.6 times as broad as costal cell (see Fletcher 2008: 112, Fig. 15). BL: 4.5–5.5 mm (male) (Distant 1917). Australia (New South Wales)	Aprivesa pronotalis (Distant)
–	Forewing brown or fuscous, with hyaline mottlings; precostal area at middle 2.4 times as broad as costal cell	2
2	Forewing with ratio of length to maximum width 1.8:1, MA relatively long (see Fletcher 2008: 111, Fig. 14). BL: 6.5 mm (male) (Muir 1931). Australia (Western Australia, South Australia, New South Wales)	Aprivesa varipennis Muir
–	Forewing with ratio of length to maximum width 2.3:1, MA relatively short	3
3	Forewing with three large hyaline spots, Cu forking about level of junction of claval veins (see Fletcher 2008: 110, Fig. 13); frons wider at widest part than long in middle line (1.5:1) (see Fletcher 2008: 112, Fig. 17). BL: 6.0 mm (male) (Melichar 1898a, b). Australia (Queensland, New South Wales)	Aprivesa exuta (Melichar)
–	Forewing with one large hyaline spot, Cu forking before junction of claval veins; frons wider at widest part than long in middle line (1.4:1) (Figs 9–13). BL: 6.0 mm (male). India (Coorg)	Aprivesa unimaculata sp. n.

#### Aprivesa unimaculata sp. n.

urn:lsid:zoobank.org:act:446BD15B-E7E8-4132-A86E-B351EEEE07BC

Figs 9 [Fig F3] [Fig F4] 23 

##### Description.

♂ (n=1), BL: 6.0 mm, FWL: 7.0 mm; ♀ (n=1), BL: 6.0 mm, FWL: 8.0 mm. General colour brown to fuscous. Vertex, frons and clypeus brown. Eye brown. Ocelli yellowish. Rostrum pallid. Pronotum and mesonotum fuscous. Thorax fuscous ventrally, marked with brown. Legs pale brown; tarsi and tips of tibiae fuscous. Abdomen fuscous ventrally, with pale brown transverse strips; pygofer fuscous. Forewing brown, with many pale brown areoles; stigma relatively large, white hyaline. Hindwing pale brown.

Head (including compound eyes) (Figs 9–12) slightly wider than pronotum. Vertex (Fig. 11) wider at anterior margin than long in middle line (5.8:1). Frons (Fig. 12) wider at widest part than long in middle line (1.4:1); disc tricarinate, with sublateral carinae shorter than central carina. Clypeus (Fig. 12) triangular, with central carina. Rostrum long, nearly reaching between hind coxae, with apical segment slightly shorter than basal segment.

Pronotum (Fig. 11) wider at widest part than long in middle line (7.1:1), punctuated beside central carina. Mesonotum (Fig. 11) large, longer than broad, tricarinate on disc, lateral carinae on each side diverging from the middle one, disunited on the anterior border. Wing venation as in Figs 13–14.

Female genitalia (Figs 15–18) symmetrical (Stroiński 2002; Liang 2003). Anal tube (Figs 15–16) relatively short and small, with apical margin rounded, nearly parallel-sided in dorsal view. Gonopophyses VIII (first valvulae) (Fig. 18) with two triangular lobes, the outer lobes saw-like, strongly sclerotised and having 7 blunt teeth on dorsal margin, and the inner lobes slightly sclerotised, with 3 blunt teeth on dorsal margin. Gonopophyses IX (second valvulae) small, degenerated. Gonoplacs (third valvulae) (Figs 15, 17) triangular with many teeth extending along ventral margin, directed mesad. Bursa copulatrix (Fig. 15) large, with two pouches, the first pouch connected to the second by a short and narrow duct, the opening directed into vagina. Genital opening singular (monotrysian), occurring between gonopophyses VIII.

Male genitalia with pygofer (Fig. 19) narrow and high, with dorsal posterior margin smoothly produced posteriorly in lateral view. Anal tube (Figs 19–20) moderately small, distinctly projected caudad, dorsally sulcate in distal half, trapezium in dorsal view, longer than wide at middle (1.3:1). Anal styles (Figs 19–20) relatively short and small. Genital styles (Fig. 19) relatively large and slender, with a long apical process, the base of inner margin curvedly produced, in profile longer than wide at middle (4.3:1). Aedeagus (Figs 21–23) cone-liked, nearly straight, partly sclerotised, symmetrical, having two pairs of caudad directed membranous processes at apex, with the inner pair longer and the outer pair slightly short.

##### Type material.

**Holotype** ♂. Ammatti, S. Coorg, S. India, xi.1982, P S Nathan (NCSU). Paratype: 1♀, S. Coorg, S. India, Ammatti, 3100 ft., v.1951, P S Nathan (NCSU).

##### Etymology.

This species is named for its forewing with one large hyaline spot.

##### Distribution.

South India (Coorg).

##### Remarks.

This species is externally similar to Aprivesa exuta (Melichar, 1898)from Australia, butcan be distinguished from the latter by the characters given in the key.

## Supplementary Material

XML Treatment for Aprivesa
